# Biochar from fungiculture waste for adsorption of endocrine disruptors in water

**DOI:** 10.1038/s41598-022-10165-4

**Published:** 2022-04-20

**Authors:** Raquel Andrade Leite Vieira, Thaisa Borim Pickler, Talita Cristina Mena Segato, Angela Faustino Jozala, Denise Grotto

**Affiliations:** 1 LAPETOX - Laboratory of Toxicological Research, University of Sorocaba, Sorocaba, Brazil; 2 LAMINFE - Laboratory of Industrial Microbiology and Fermentation Process, University of Sorocaba, Sorocaba, Brazil

**Keywords:** Environmental sciences, Chemistry

## Abstract

The agricultural residues are ecofriendly alternatives for removing contaminants from water. In this way, a novel biochar from the spent mushroom substrate (SMS) was produced and assessed to remove endocrine disruptor from water in batch and fixed-bed method. SMS were dried, ground, and pyrolyzed. Pyrolysis was carried out in three different conditions at 250 and 450 °C, with a residence time of 1 h, and at 600 °C with a residence time of 20 min. The biochar was firstly tested in a pilot batch with 17α-ethinylestradiol (EE2) and progesterone. The residual concentrations of the endocrine disruptors were determined by HPLC. The biochar obtained at 600 °C showed the best removal efficiency results. Then, adsorption parameters (isotherm and kinetics), fixed bed tests and biochar characterization were carried out. The Langmuir model fits better to progesterone while the Freundlich model fits better to EE2. The Langmuir model isotherm indicated a maximum adsorption capacity of 232.64 mg progesterone/g biochar, and 138.98 mg EE2/g biochar. Images from scanning electrons microscopy showed that the 600 °C biochar presented higher porosity than others. In the fixed bed test the removal capacity was more than 80% for both endocrine disruptors. Thus, the biochar showed a good and viable option for removal of contaminants, such as hormones.

## Introduction

The human endocrine system is an integrated structure of multiple organs able to synthesize and release chemical messengers, named hormones. Hormones perform functions in target cells near or far from the release site. The endocrine system coordinates and integrates cellular activities throughout the body, such as the regulation of sodium and water balance, energy balance, regulation of development, growth, reproduction, and the aging process. When the correct combination of hormone and target cell receptor occurs, the physiological response of the hormonal action happens^1^.

However, the receptors do not have the characteristic of only making connections with specific hormones. Receptors are capable to bind other chemicals with a compatible chemical structure to the same receptor. Chemicals are responsible for making changes related to the endocrine system are named endocrine disrupters. These substances are microcontaminants originated from anthropic actions, which are released into the environment in trace concentrations (µg/L–ng/L), affecting the ecosystem and human health^2,3^.

According to the World Health Organization (WHO), endocrine disruptors “are substances or mixtures of exogenous substances, which alter the normal functions of the endocrine system, causing adverse effects on the individual, his progeny or populations of organisms exposed to such substances”^4^. The presence of these contaminants in water implies a real health risk. Studies and improvements in water treatment systems are crucial due to the final route of those contaminants ends up in water resources and water supply systems^5^.

17α-ethinylestradiol (EE2) and progesterone are widely used in the pharmaceutical industry, as contraceptives, and in the dairy industry, as hormonal cycling induction of cattle. Both are found in environmental by the contamination of water, affecting the ecosystem^3,6,7^.

In Brazil and in many countries, there are still no environmental laws regarding endocrine disruptors maximum concentrations in rivers, or potable water. Several studies report concentrations of EE2 in different regions from Brazil, e.g., between 0.194 ng/L and 48.2 µg/L^8–11^, with the concentrations varying throughout the year. According to Cunha et al.^12^, concentrations above 0.035 ng/L are already sufficient to cause adverse effects on the induction of vitellogenin in male fish, affecting the reproduction system of these animals.

Biochar have been used in contaminated water as a solution for the removal of microcontaminants. Biochar are solid materials rich in carbon, obtained through the process of heating biomass from the industrial and agricultural wastes, called pyrolysis. It is in pyrolysis that the formation of pores occurs^13,14^. The properties acquired in the pyrolysis process will determine the increase in adsorption capacity, such as surface area, charges, chemical functionality, and the amount of pores^14^. Biochar seems a sustainable alternative to two problems: viable routing to the waste from agricultural production, and water treatment^13^.

Spent mushroom substrate (SMS) is a general residue from fungiculture; it is a promising residue that can be used as biochar. Kulshreshtha^15^, reported an annual consumption of edible mushrooms over 4 kg/year/per capita worldwide in 2012. In this way, the production of mushroom residues rises in the same direction. In a previous study, it was reported that 1 kg of edible mushrooms can generate 5 kg of spent mushroom substrate^16^.

There are some studies regarding mushrooms as biosorbents of metals^17,18^ and dies^19^. However, there are not so many studies regarding endocrine disruptor biosorption by biochar. Some of them investigated the endocrine disruptor removal by adsorbent such as rice husk biomass in water^20^, biofertilizer material with rice husk or soybean hull^21^, graphene-like synthesized using lotus seedpod^22^, lotus seedpod^23^ and carboxymethyl cellulose^24^. Our team have initiated an investigation related to the bioremediation of the EE2 using mushroom disposal^25^; nevertheless, in the initial study no charcoal was produced. In this way, the novelties of this work were (i) to produce different biochar from spent mushrooms substrate, and (ii) to use the biochar in water experimentally contaminated with endocrine disruptors.

## Results

### Pilot batch test

The pilot batch test was performed to identify the biochar with the higher removal capacity. In Table 1 is presented the value of removal capacity for the three biochar at different temperatures.

**Table 1:** Removal capacity of the biochar in the pilot batch test for the three different pyrolysis temperatures.

Temperature (°C)	Time (min)	EE2	Progesterone
		% Removal	% Removal
250	10	71.06	16.24
	30	77.36	27.88
	60	78.76	30.82
450	10	70.95	19.65
	30	71.53	20.24
	60	73.05	32.00
600	10	93.54	94.04
	30	95.54	96.68
	60	95.87	97.26

The batch test was an important analysis to optimize further processes. It was identified that the biochar pyrolyzed at 600 °C had the best performance for removal both hormones.

### Biochar characterization

Table 2 shows the concentration of the chemical elements in SMS before and after the pyrolysis process at 600 °C, assessed by X-ray diffraction (XRF). It is observed an increase in the percentage of many elements due to the process of heating, reducing the organic matrix (CHN), and consequently concentrating other chemical elements. SMS presented 96.59% CHNS, and the percentage presented reduced in biochar (79.55%).

**Table 2:** Concentration of chemical elements in samples of dried spent mushroom substrate (SMS) and biochar after pyrolysis at 600 °C.

Compounds*	SMS (%)	Biochar (%)
Organic Material (C_6_H_10_O_5_)	96.5	79.4
Co	0.004	0.005
Mg	0.355	3.368
Al	0.002	0.151
Si	0.390	1.286
S	0.094	0.146
K	0.606	3.783
Ca	0.924	5.473
Mn	0.050	0.275
Fe	0.085	0.462
Cu	0.001	0.009
Zn	0.008	0.046
Rb	0.003	0.018
Sr	0.004	0.026

*The lowest concentrations of compounds were not shown in the table.

**Figure 1:**
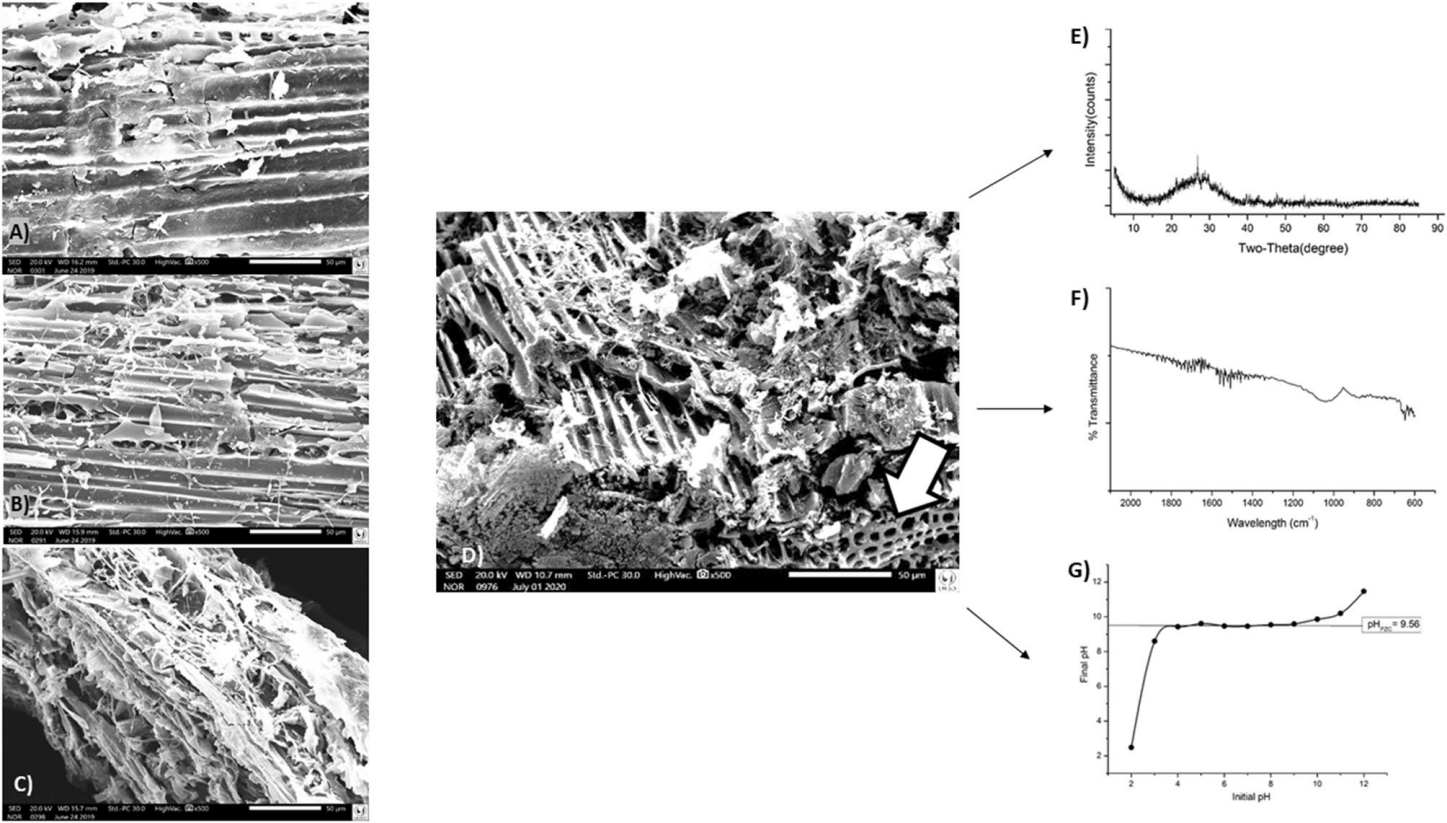
Images of scanning electron microscopy of Spent Mushroom Substrate (SMS), before pyrolysis (**A**). Biochar from SMS after pyrolysis at (**B**) 250 °C, (**C**) 450 °C and (**D**) 600 °C. In (**E**) X-ray diffraction patterns of the biochar at 600 °C; in (**F**) Fourier Transform Infrared (FTIR) spectra of the biochar at 600 °C; in (**G**) Behavior of pH for the pH_PZC_ test for the biochar pyrolyzed at 600 °C. The white arrow shows the blisters in the biochar at 600 °C.

Biochar surface characterization was performed by SEM, showed in Fig. 1A–D. SMS was evaluated before and after the pyrolysis process. The structural changes—induced by thermal treatment, and the formation of pores, that increase the surface area, were identified.

It is possible to observe that the SMS before pyrolysis had a more homogeneous structure, with longitudinally patterned recesses. With the pyrolysis processes at 600 °C, the break of the SMS is clearly visualized, and the blisters were exposed, probably increasing the surface area and consequently improving contaminants removal^26^. Also, at 600 °C the pores formation with sizes from 1.5 to 5.0 µm were obtained. The SMS before pyrolysis did not showed pores. The pyrolysis process at 250 and 450 °C with 1 h residence time proved not to be sufficient for porous formation.

The BET surface area of the biochar was 246 m^2^/g. The XRD and FTIR analyses are showed in Fig. 1E,F, respectively, in which the material composition and the structure of the biochar are presented.

The pH_PZC_ was performed with the biochar pyrolyzed at 600 °C. The mean value of pH_PZC_ was 9.56 ± 0.14 (Fig. 1G). Thus, all solutions were adjusted to pH 9.56. However, for extra-tests, the adsorption capacity was also evaluated without pH adjustment, and the results remained at the same removal percentage for solutions with pH up to 4.3, demonstrating the biochar’s robustness.

### Removal efficiency and kinetics

**Figure 2:**
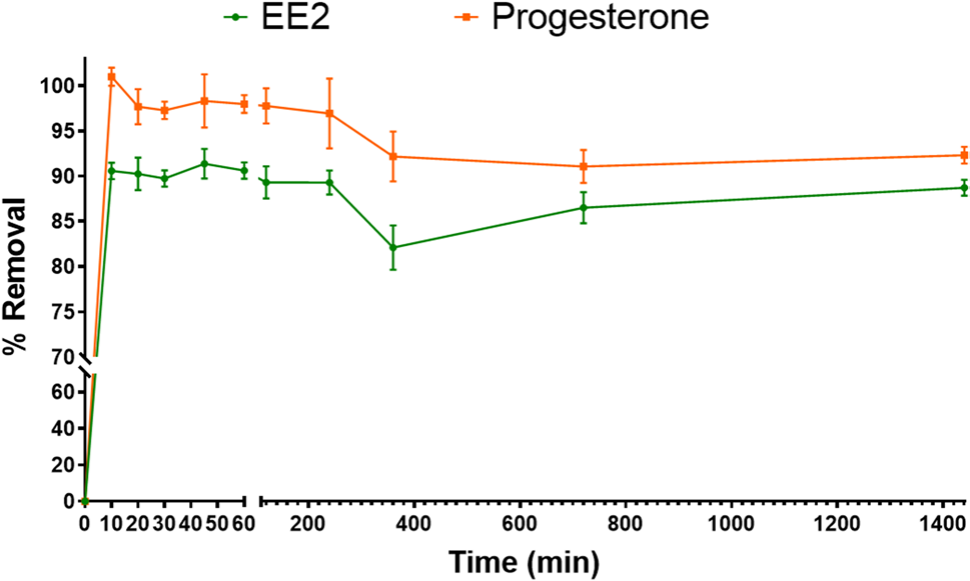
Removal efficiency over time of 17α-ethinylestradiol (EE2) and progesterone.

Considering the fixed concentrations of EE2 and progesterone, 1.0 and 10 mg/L, respectively, the biochar removal efficiency, over time, is presented in Fig. 2. There was a high removal rate, 89% for EE2 and 91% for progesterone, in the first 10 min of contact between adsorbent and contaminants. The results show that initially, the adsorbent removes a large part of the contaminants due to its free surface area for adsorption.

**Figure 3:**
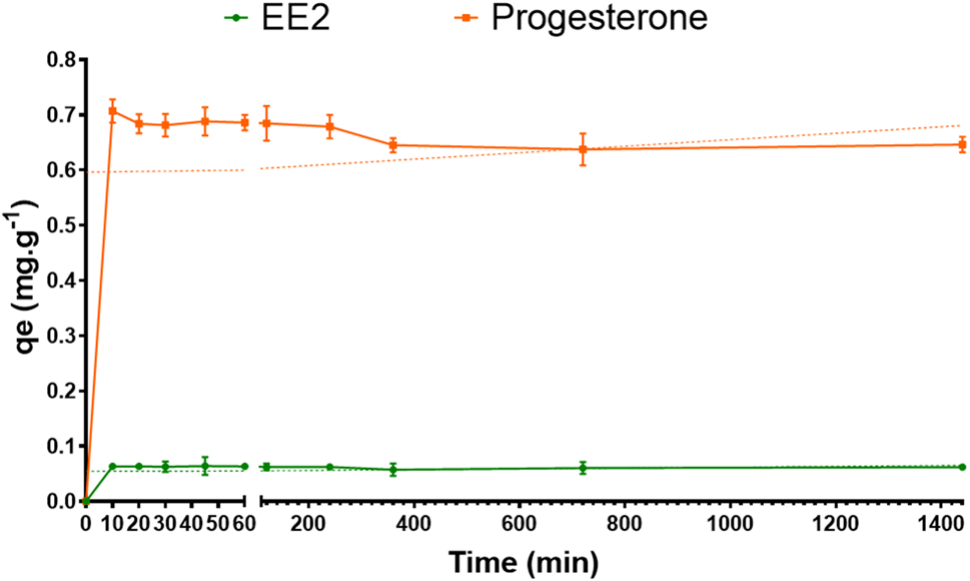
Kinetic adsorption of the biochar for 17α-ethinylestradiol (EE2) and progesterone.

Figure 3 shows the kinetic adsorption for both disruptor endocrines. The adsorption of EE2 onto the biochar showed two distinct phases: a rapid phase over the first 10 min and a second phase of equilibrium, during until 1440 min. According to the experimental data, the determination coefficient (R^2^) of the pseudo-second-order model (0.9392) was higher than those of the pseudo-first-order model (0.4544). The same was observed to progesterone: a rapid phase of adsorption in the first 45 min and a second phase of equilibrium. On the other hand, R^2^ of the pseudo-first-order model (0.8651) was higher than those of the pseudo-second-order model (0.225).

By comparing the R^2^ of the endocrine disruptors, data indicated that the pseudo-second-order kinetic model can be considered for EE2 adsorption onto the biochar, and the adsorption may be due to chemisorption, adsorption processes involving chemical bonding between the adsorbate and the functional groups of the biochar.

### Adsorption isotherms

The adsorption isotherm results for both endocrine disruptors were analyzed by the Langmuir and Freundlich models. The parameters of the isotherm are showed in Table 3.

**Table 3:** Parameters of the adsorption isotherm following Langmuir and Freundlich models for 17α-ethinylestradiol (EE2) and progesterone, where R^2^ is the determination coefficient, *n* is the heterogeneity parameter and K_L_ and K_F_ are the constants of Langmuir and Freundlich models, respectively.

	Parameter	EE2	Progesterone
Langmuir model	*n*	0.016	0.001
	K_L_ (mg/L)	0.443	0.4334
	R^2^	0.9268	0.7083
Freundlich model	*n*	1.1073	0.158
	K_F_ (mg/g)	48.323	122.49
	R^2^	0.9478	0.3405

Langmuir and Freundlich isotherm models showed an exceptional value for R^2^ for EE2, while R^2^ for Langmuir model was suitable for progesterone and for Freundlich model it was inadequate. In general, we can assume, based on R^2^, that the Langmuir model fits better to progesterone while the Freundlich model fits better to EE2. Considering constant values, the higher the K value, the more affinity between the absorbing molecules and the adsorbate. Thus, the Freundlich isotherm model showed the best results for both endocrine disruptors.

Regarding the heterogeneity parameter, when it is between 1 and 10, it indicates favorable adsorption, that means the interactions between adsorbent and adsorbate are stronger when the n value is higher. Here, the Freundlich n values was 1.107 for EE2, suggesting favorable adsorption.

Maximum absorption capacity (*q*_*max*_) is a relevant parameter from the Langmuir isotherm model. EE2 reached a *q*_*max*_ of 138.98 mg EE2/g biochar, while the *q*_*max*_ of progesterone was 232.64 mg/g biochar.

### Fixed bed

Fixed bed process is studied considering mainly porosity and packing density. The apparent density was 1.23 g/cm^3^ and an apparent volume was 2.14 g/cm^3^. The column calculated volume was 10 cm^3^, with a packing density of 0.26 g/cm^3^ and a porosity of 0.97.

**Figure 4:**
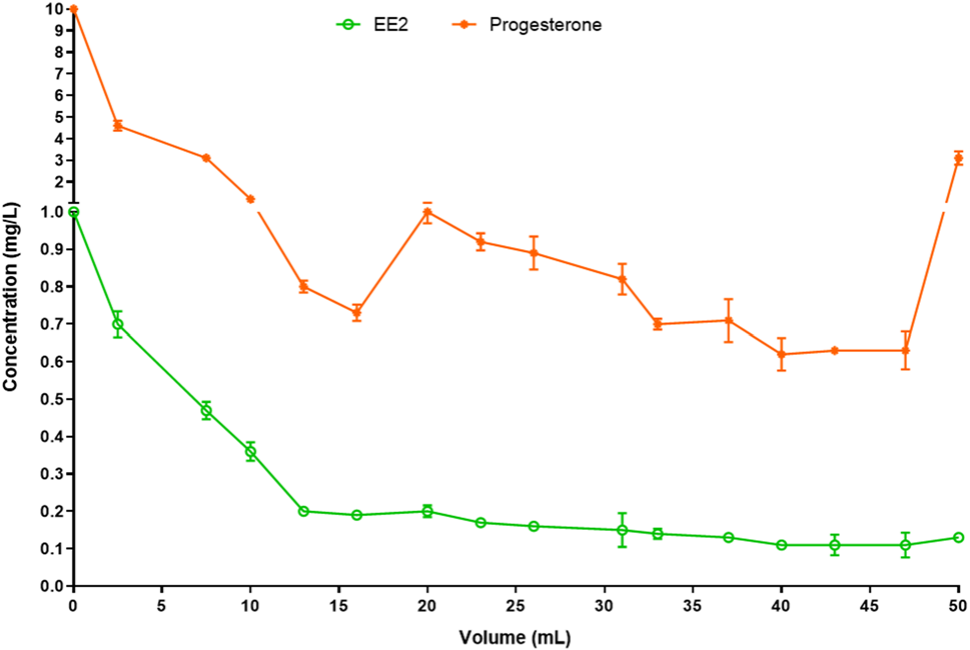
Breakthrough curves of the pyrolyzed biochar for 17α-ethinylestradiol (EE2) and progesterone.

The rupture curves for EE2 and progesterone are shown in Fig. 4. It was not possible to calculate the break point for EE2, once the removal of this endocrine disruptor continued to occur even after the entire volume of the solution had been passed through the column. On the other hand, the break point for progesterone starting at 46.8 mL. However, the removal was still not null at this point since the final concentration was lower than the initial one.

## Discussion

In this research, novel biochar from fungiculture waste were developed as a material for the adsorption of emerging chemical compounds, such as hormones. The biochar produced at 600 °C with 20 min residence time showed formation of pores and recesses. This finding justifies the results from the pilot batch test, once the increase of the surface area led to the consequent higher removal of hormones.

The biochar produced at 600 °C presented defined and identifiable tubes, with different geometry. Also, blisters were detected both intrinsically (within the pores) and externally on the surface. These blisters can result from incomplete or non-bursting bubbles generated by the escaping volatiles during the pyrolysis process of the organic material. Broken pieces of this structure can be seen dispersed on the surface of the biochar suggesting the fragile nature^19^. The large surface area, 246 m^2^/g, of the material makes it a good candidate for the adsorption of several other contaminants.

Regarding biochar characterization, results from the XRF analysis showed a 17.7% decrease in organic material in biochar after the pyrolysis process. And the elements Mg, K, Ca, Mn, Si, Al and Fe have risen with the increase in temperature, as organic material transformed into hydroxides. This fact explains the high pH value in the PZC test. The concentration of the elements, in general, increases because the of loss of organic material during pyrolysis^18^. Naturally, minerals are present on all types of biomass, and moreover some minerals can be deposited on the biomaterial during the treatment of the sample^27^. In the case of SMS process, the material had been in contact with the stove, the knife mill, the muffle and the crucibles, that previously were used in others tests and could contain residue of Al, Si, Fe and Mg.

SMS had higher amount of cellulose in its composition compared to the biochar. Pyrolysis decreased the organic material, but this process contributed for the formation of micro and mesoporous structures, providing more active site for adsorption of hormones. This result supports the great surface area of the biochar, which was higher than those reported in other studies, with modified and non-modified biochar^28–30^.

In X-ray diffraction (Fig. 1E), peaks with the highest intensities were 2θ = 21° and 2θ = 26.7°, related to the presence of quartz compound. The peaks at 2θ = 29.5° and 2θ = 30.4° confirmed the presence of CaCO_3_ and Ca_3_(PO_4_)_2_, respectively^30^. In the FTIR spectra (Fig. 1F), the bands around 1000–1100 cm^−1^ are related to the C–O and C–O–C stretching vibrations associated to phenol, ester and alcohol groups; here, the functional groups with oxygen-containing are originated from the presence of cellulose. The bands at 1688 and 1632 cm^−1^ indicated the C=O and C=C groups, respectively. These structures could provide π-electrons that contribute to the adsorption process.

Functional groups have important effects in the adsorption capacity and on the physicochemical properties. Solid sorbents, when in contact with a solution with pH below pH_PZC_ value, present a positively charged surface, and thus anions are attracted to balance the positive charges, generating an increase of the solvent affinity. In solutions with a pH higher than pH_PZC_ value, the adsorbent surface is negatively charged, thus attracting cations. The difference in charges between adsorbent and solution is an important factor for efficient adsorptio_n2_^3^. Considering the biochar produced at 600 °C, the alkalinity is related to its inorganic minerals, and improves the adsorption of the compound positively charged. Another point for pH_PZC_ was that the biochar was not washed after the pyrolysis process, which may have caused the increase of pH value. Thus, in alkaline pH the best adsorption of contaminants was observed. It has been reported that 10.4 is the pKa of EE2^31^.

Liu et al.^32^ used rice husks to produce biochar to adsorb medications such as tetracycline from water. They obtained a pH_PZC_ of 8.0, which means the best adsorption in alkaline solutions. Guilarduci et al.^33^ conducted a study with activated carbon for phenol compounds removal. pH_PZC_ test helped to conclude pH 10.0 was the one with the best result, due to in high pH values alkaline groups present on the coal surface are ionized, becoming available for the formation of donor-receptor complexes.

Time is an important variable in the analysis once it determines the behavior of the adsorbent at a given concentration of adsorbate. Over time, the removal percentage begins to decline slowly. Generally, the adsorption occurs first on the surface of the material, follow by intraparticle adsorption, which occurs slowly^34^. In addition, desorption process can occur, that is, the adsorbed contaminant returns to the aqueous medium. From 40 to 240 min, in both contaminants, there was linearity in the removal, with removal between 86 and 88%, followed by a drop.

The pseudo-first order model is based on the supposed that adsorption rate is controlled by the adsorbate diffusion, and the pseudo-second order model relies on the supposed the rate-limiting step is the chemical adsorption step due to surface adsorption interactions^30^. Both endocrine disruptors showed two distinct phases of adsorption: a rapid phase over and a second slow phase until reaching equilibrium. For EE2, the rapid phase lasted 10 min, and for progesterone, 45 min. According to the experimental data, we suggest the pseudo-second-order model can be considered to EE2, while the pseudo-first-order kinetic model represents progesterone.

Adsorption isotherms were evaluated to express the distribution or equilibrium ratio of the remaining endocrine disruptors in the solution and the amount of adsorbate adsorbed by the biochar. In addition, adsorption isotherm reveals information about the maximum capacity of adsorption of the biochar (q_*max*_). Comparing to other studies, q_*max*_ obtained in the Langmuir model was much higher than those reported in literature^35^. Table 4 shows other biochar and other contaminants, for comparative purpose.

**Table 4:** Studies with different adsorbents for removal organic contaminants.

Adsorbents	Contaminant	q_max_ (mg/g)	References
Bamboo biochar	Chloramphenicol	2.1	Fan et al.^36^
Rice husk biochar	Tetracycline	58.8	Liu et al.^32^
Corn straw biochar	Atrazine	11.6	Zhao et al.^37^
Paper mill sludge biochar	Pentachlorophenol	50.0	Devi, Saroha^38^
Rice husk biomass	Estrone	2.698	Honorio et al.^20^
	17β-estradiol	1.649	
	Estriol	0.979	
Lotus seedpod biochar	17β-estradiol	From 120.9 to 147.1	Liu et al.^23^
Lotus seedpod and potassium ferrate	17β-estradiol	From 116.1 to 121.2	Liu et al.^22^
Champignon (*Agaricus bisporus*)	17α-etinil estradiol	5.62	Menk et al.^25^
Shiitake (*Lentinula edodes*)		18.95	

It is a quite difficult to compare the adsorption capabilities of our material to others, due to inconsistency and lack of data. Most of the tests had different adsorption conditions, they were conducted in batch or columns, and mainly different types of adsorbent preparation and contaminants^39^.

Menk et al.^25^ evaluated the adsorption capacity of some mushrooms in their natural form (shiitake and champignon), as well as the SMS, for possible use as adsorbers for EE2 and paracetamol drug. The SMS presented a R^2^ 0.97906 for the Sips model and q_*max*_ 0.31 mg EE2/g substrate. At the moment, the use of a biochar from SMS for hormones adsorption has not been reported. And based on the findings reported by Menk et al.^25^, SMS achieved a significant improvement when submitted to pyrolysis, once q_*max*_ for EE2 was upgraded to 138.98 mg EE2/g biochar. The q_*max*_ value is related to the surface, in which the higher values refer to smaller particles and higher surface area, increasing the adsorption. Adsorption values from biochar at 250 and 450 °C were lower than those founded by Menk et al.^25^, due to there was no effective formation of pores at those temperatures and because the degradation of functional groups, as lignin. Moreover, in a study with a corn straw biochar, the authors reported a q_*max*_ of 1.696 ± 0.202 mg EE2/g corn biochar^40^. Compared to our findings, the q_*max*_ for EE2 our biochar is about 85 times higher than those reported by Guo et al.^40^.

Porosity as well as packing density are important parameters for studying the behavior of fixed bed process. Packing density characterizes the bed regarding cavities among particles. Thus, when the system has a high packing density and small particles, there is a great loss of pressure, that is, a loss of flow force. In systems with low packing density and small particles, preferential paths are formed, that is, paths that present an easier passage for the eluent^41^.

According to Wang et al.^42^, the fluid tends to move to regions with greater empty space, thus forming the preferred paths. Therefore, it is important to consider the arrangement of the fiber in the column (spatial arrangement), in order to avoid the occurrence of such paths. Thus, a higher packing density does not necessarily mean a greater mass transfer since the dead zones (from the preferred paths) increase with the packing density. In a filling column system in which preferential paths are present, it implies a shorter residence time, that is, a less effective interaction between the solid and the hormone^43^.

The rupture curve is a data used to describe the contaminant concentration by the volume of treated contaminated solution. In this way, it is possible to identify the breaking point of the process, which means the exhaust volume of the adsorbent^44^.

In some cases, the adsorbents have a large adsorption capacity in the fixed bed, leading to longer time to reach the breakthrough volume and the process exhaustion to the treated volume. In this case, the total volume for establishment of the mass transfer zone, in which the final concentration is equal to the initial one, can not be estimated. And this finding occurred in our study. The total volume of the mass transfer zone was not discovered in EE2 fixed bed test. Regarding progesterone, it is possible to suggest, observing Fig. 4, that the desorption process seems to start in 240 min, at 46.8 mL. However, the final concentration was still lower to the initial one.

According to Barbosa^41^, the smaller mass transfer zone, the closer to ideality of process, that means the removal is favorable. After this time, bed saturation would occur. We suggest our biochar takes longer time for the formation of the primary adsorption zone, which is the space between the rupture point and the point of exhaustion.

## Conclusion

SMS is an ideal raw material for the preparation of biochar. The big surface area and the numerous microporous structures of the SMS biochar pyrolyzed at 600 °C were efficient for hormones adsorption.

The adsorption of EE2 and progesterone can be described as chemisorption and monolayer adsorption process, which is related to the functional groups present in the biochar after pyrolysis. The Langmuir model fits better to progesterone while the Freundlich model fits better to EE2. The maximum adsorption capacity for both endocrine disruptors was much higher than the literature. Thus, using residues from fungiculture as biochar can be an alternative for water treatment with circular economy.

## Methodology

### Biochar preparation

SMS used in this study was supplied from the commercial company Yuri Cogumelos, located in Sorocaba city. The SMS from *Lentinula edodes* mushroom cultivation mainly consisted in a mixture of wheat and rice bran (14%), eucalyptus sawdust (21%), and water (65%). SMS was dried in an oven at 105 °C for 24 h. Then the samples passed through the knife mill (Marconi—MA340 Series 0004201) and later through the granulometric sieve at 80 mesh granulometry was used.

Pyrolysis process was applied to produce biochar from the SMS samples. Hence, 3 g of SMS (80mesh) were weighed in each porcelain crucibles and placed inside the muffle furnace (SP Labor, 1200) at different temperatures 250, 450 and 600 °C.

At 250 and 450 °C, longer pyrolysis was performed, the residence time was 1 h; and at 600 °C the residence time was 20 min. The residence time of 20 min was determined based on a pilot test run for 1 h at 600 °C, in which the material was converted into ashes, making its use impossible. After the pyrolysis process, the three samples were weighed and stored in a plastic container at room temperature.

### Pilot batch test

The pilot batch test was run to identify the biochar with the best hormone removal capacity. For this reason, a mass of 0.5 g of each SMS biochar (made at 250, 450, and 600 °C) was added, distinctly, in Erlenmeyer flasks with 35 mL of progesterone solution at 10 mg/L or 17α-ethinylestradiol (EE2) at 1 mg/L; both prepared in methanol:water 20:80 v/v. The Erlenmeyer flasks were kept under constant agitation at 100 rpm, in an orbital shaker (Nova Técnica, NT 715). The pilot batch test run with three collection times 10, 30, and 60 min, at 25 °C. The temperature of 25 °C was chosen once it is considered the standard ambient temperature, in which any water treatment plant could act.

These concentrations are purposefully higher than those found environmentally. Analytically, removal efficiency and isotherm need to be run in high concentrations to saturate the aqueous medium and thus bioremediation is capable to be studied.

After each time collection, the sample was filtered, and the hormones quantifications were performed using the High-Performance Liquid Chromatography (HPLC) technique. First of all, a method for simultaneous determination of EE2 and progesterone was validated, using HPLC. The retention time of EE2 occurred in 1.4 min and for progesterone in 2.4 min, and the peak amplitude value was used to perform the analytical calibration curve.

### Biochar characterization

The biochar with the best adsorption result in the pilot test was characterized. The surface of the samples was analyzed by Scanning Electron Microscopy (SEM) (JEOL, JSM-IT200A) at 15 keV. The metallization process with gold (JEOL, Smart Coater) was carried out for a better sample scan. X-ray Fluorescence (XRF) (Malvern Panalytical, Epsilon1) was performed to analyze the composition of the material.

The Fourier transform infrared spectroscopy (FTIR) (IRTracer-100 Shimadzu) using attenuated total reflectance (ATR) analysis to determine the functional groups in the biochar and the spectra were generated in the range of 2000 to 400 cm^−1^.

The superficial area of the biochar was estimated by the analyzer Quantachrome NOVA 4200e by X-Ray Diffraction (XRD) (Shimadzu XDR7000), using a CuK α tube (λ = 1.5406 A) at 40 kV and 30 mA. The scan range was from 5° to 85° with a step size of 0.02° per second (raw data in the Supplementary Material).

### Determination of point of zero charge (pH_PZC_)

The biochar with the best adsorption result in the pilot test was applied to pH_PZC_. The PZC analysis aims to determine the pH value in which surface of the adsorbent material, defining whether the adsorption is effective. In the experiment, PZC was made by measuring 11 pH values, ranging from 2.0 to 12.0^24,45^. The procedure consisted of adding 20 mg of biochar in 20 mL of 0.1 mol/L aqueous NaCl solution, under 11 different initial pH points, adjusted with HCl or NaOH 0.1 mol/L. After 24 h at 100 rpm agitation and 25 ± 2 °C, the final pH value was measured. The pH_PZC_ corresponds to the point at which the pH value is constant after the sample has reached equilibrium. All solutions were adjusted to pH found in the PZC test.

### Adsorption kinetics

The biochar with the best adsorption result in the pilot test was used to perform the adsorption kinetic. A mass of 0.5 g of biochar was added to 35 mL of the hormone solution (1 mg/L of EE2 and 10 mg/L of progesterone), pH adjusted according to PCZ. Erlenmeyer flasks (n = 10) were placed in a shaker incubator (New Technique—model NT 715) for 24 h, 100 rpm at 25 ± 2 °C. Each Erlenmeyer was a withdrawal time: 10, 20, 30 45, 60, 120, 240, 360, 760, and 1440 min. All samples were vacuum filtered (Millipore—Sterifil) with a simple filter to retain the solid part (biochar) of the aqueous medium. Then residual aqueous solutions were stored at 5 °C until HPLC analysis.

### Adsorption isotherm

The biochar with the best adsorption result in the pilot test was used to perform the adsorption isotherm. Isotherm relates the amount of substance adsorbed and the equilibrium concentration of a solution at a given constant temperature. An amount of 0.5 g of biochar was added to 35 mL of the hormone solution in five different concentrations: 5.0, 2.5, 1.0; 0.8; 0.6; 0.4 and 0.2 mg/L for EE2 and 50, 25, 10, 8, 6, 4, 2 mg/L for progesterone. Samples were shaken for 24 h at 25 ± 2 °C, 100 rpm in the shaker incubator (New Technique—model NT 715). After that, samples were vacuum filtered and stored at 5 °C until HPLC analysis.

### Fixed bed

The fixed bed test was also performed with the biochar with the best adsorption result in the pilot. A chromatographic column was used, with a diameter of 20 mm and 22 cm height; a porous plate and a Teflon tape. The column was filled with 2 g of the biochar, as a fixed bed. The EE2—progesterone solution passed through the column at a flow rate of approximately 3.4 mL/min, at 25 °C. The post-column samples were collected at 1, 2, 3, 4, 5, 10, 20, 30, 40, 50, 60, 120, 180 and 240 min. The aqueous samples were stored at 5 °C until HPLC analysis.

### HPLC analysis

The analytical technique of HPLC was applied for the hormone’s quantification in batch tests, adsorption kinetic, isotherm, and fixed bed. A C18 reverse-phase column (Thermo Scientific) was used, 125 mm × 4.60 mm, with 5 μm particle size. The column was thermostatic at 37 °C. Some trials in the flow rate, running time, as well as in mobile phase concentration were run to validate a simultaneous analysis of the two hormones EE2 and progesterone, based on the methodology of Unruh (2011)^46^. Tests were analyzed in duplicate for greater reliability of results.

A simultaneous quantification of EE2 and progesterone was developed to improve the investigation of the residue concentrations in all the tests. Thus, the chromatographic conditions validated were flow rate 1.6 mL/min, 4 min running time, 20 μL injection volume, detection at 202 nm, mobile phase composed of 70% acetonitrile standard HPLC (Sigma-Aldrich) and 30% Milli-Q water, in an isocratic system. Analytical curves were performed using the amplitude of the peaks. The concentrations for EE2 were 10, 1.0, 0.5, 0.3, 0.1 and 0.001 mg/L and for progesterone were 100.0, 10.0; 5.0; 3.0; 1.0 and 0.1 mg/L. The equation of the line (y) and the determination coefficient (R^2^) were calculated using Microsoft Excel software. The curve was performed in duplicate, and the arithmetic average of the amplitude values was used. The EE2 equation obtained by the least squared regression was y = 0.0408x − 0.0059, with R^2^ 0.9986, and progesterone equation was y = 0.0013x + 0.0019, with R^2^ 0.9996.

### Data analysis

Data were expressed as mean ± standard deviation, or percentage of sorption. Data analyses were performed in the software Microsoft Office Excel and GraphPad Prism.

The equations used for adsorption efficiency were:

1 $$\% Removal \,efficiency: \frac{(Co-Ce)}{Co}\times 100,$$

2 $$Qe= \frac{Co-Ce }{m} \times V,$$

where *Co* and *Ce* (mg/L) were the initial and equilibrium concentrations of endrocrine disruptors, respectively; *m* (g) was the amount of the biochar; *V* (mL) was the volume of the solutions and Qe was the adsorption capacity.

The kinetics results were analyzed by pseudo-first and pseudo-second-order kinetics models, and the equations used were^47^:

3 $$\mathrm{ln}\left(Qe-Qt\right)=lnQe-{K}_{1}t,$$

4 $$\frac{t}{Qt}= \frac{1}{{K}_{2}{Qe}^{2}}+ \frac{t}{Qe},$$

in which *Qt* (mg/g) was the adsorption capacity at time t (min), and k_1_ and k_2_ were he rate constants for pseudo-first and pseudo-second-order adsorption, respectively.

Moreover, the adsorption isotherm results were analyzed by the Langmuir and Freundlich models, and the data were fit in Origin 9.0.

## Supplementary Information

Supplementary Information.

## Data Availability

Requests for materials should be addressed to D.G.
