# Supplementary material for: Biochar from fungiculture waste for adsorption of endocrine disruptors in water
Source: Sci Rep. 2022 Apr 20;12:6507. doi: 10.1038/s41598-022-10165-4 (PMC9021278; doi:10.1038/s41598-022-10165-4)

*** Basic Data Process ***

Group : CA-IQ
Data : P210509_AM-1076-21

Strongest 3 peaks

no.	peak no.	2Theta (deg)	d (A)	I/I1	FWHM (deg)	Intensity (Counts)	Integrated Int (Counts)
1	18	26.8333	3.31982	100	0.82670	71	6134
2	19	28.8200	3.09533	69	0.00000	49	0
3	20	29.5400	3.02150	68	1.02660	48	2813

Peak Data List

peak no.	2Theta (deg)	d (A)	I/I1	FWHM (deg)	Intensity (Counts)	Integrated Int (Counts)
1	6.7000	13.18212	4	0.04000	3	15
2	7.6750	11.50956	4	0.03000	3	10
3	9.8100	9.00895	7	0.10000	5	84
4	11.2350	7.86929	4	0.03000	3	13
5	12.1050	7.30560	10	0.19000	7	117
6	14.2400	6.21471	8	0.24000	6	160
7	15.2100	5.82049	4	0.06000	3	30
8	16.6400	5.32337	6	0.04000	4	23
9	17.4700	5.07228	4	0.06000	3	22
10	18.0000	4.92411	4	0.04000	3	21
11	19.8400	4.47139	14	0.44000	10	435
12	21.2650	4.17487	41	0.81000	29	1592
13	22.0800	4.02258	27	0.00000	19	0
14	22.6600	3.92091	37	0.00000	26	0
15	23.9400	3.71409	44	0.00000	31	0
16	25.1200	3.54223	48	0.00000	34	0
17	25.9800	3.42689	49	0.00000	35	0
18	26.8333	3.31982	100	0.82670	71	6134
19	28.8200	3.09533	69	0.00000	49	0
20	29.5400	3.02150	68	1.02660	48	2813
21	31.5000	2.83782	37	1.56000	26	1866
22	32.8800	2.72180	18	1.28000	13	775
23	34.3050	2.61193	13	0.37000	9	222
24	39.7150	2.26771	21	0.57000	15	440
25	40.5300	2.22397	17	0.52000	12	363
26	41.2000	2.18934	4	0.08000	3	30
27	42.8900	2.10690	17	0.98000	12	498
28	43.4800	2.07967	11	0.80000	8	266
29	44.2400	2.04569	4	0.16000	3	48
30	45.6900	1.98408	3	0.10000	2	17
31	47.6600	1.90657	21	0.48000	15	405
32	48.6500	1.87006	14	0.74000	10	382
33	50.3300	1.81151	8	0.42000	6	133
34	51.4100	1.77596	6	0.18000	4	72
35	53.0000	1.72636	8	0.12000	6	70
36	54.1200	1.69325	11	0.56000	8	204
37	54.8450	1.67257	18	0.51000	13	348
38	56.5600	1.62586	4	0.04000	3	24
39	58.0300	1.58812	10	0.10000	7	102
40	59.1200	1.56141	4	0.04000	3	16
41	60.5650	1.52757	4	0.17000	3	59
42	61.6000	1.50437	4	0.04000	3	23
43	63.1900	1.47029	11	0.26000	8	125
44	65.2500	1.42876	4	0.18000	3	43
45	66.3500	1.40772	3	0.14000	2	22
46	67.6300	1.38415	6	0.14000	4	82
47	68.5800	1.36728	7	0.24000	5	154
48	69.6300	1.34921	6	0.26000	4	109
49	73.6800	1.28473	6	0.24000	4	66
50	74.9900	1.26550	4	0.18000	3	63
51	76.0000	1.25117	6	0.28000	4	172
52	76.6000	1.24286	7	0.00000	5	0
53	78.0600	1.22323	4	0.12000	3	43

peak no.	2Theta (deg)	d (A)	I/I1	FWHM (deg)	Intensity (Counts)	Integrated Int (Counts)
54	78.9100	1.21217	7	0.30000	5	121
55	79.6000	1.20339	3	0.12000	2	53
56	81.7700	1.17685	11	0.42000	8	324
57	83.9100	1.15220	8	0.34000	6	118
58	84.5650	1.14494	7	0.15000	5	69

*** Basic Data Process ***

```
# Data Infomation
          Group                : CA-IQ
          Data                 : P210509_AM-1076-21
          Sample Nmae          :
          Comment              : OPER_DEBORAH-SIMONI
          Date & Time          : 05-17-21 08:02:59

# Measurement Condition
    X-ray tube
          target               : Cu
          voltage              : 40.0 (kV)
          current              : 30.0 (mA)
    Slits
          Auto Slit            : not Used
          divergence slit      :   1.00000 (deg)
          scatter slit         :   1.00000 (deg)
          receiving slit       :   0.15000(mm)
    Scanning
          drive axis           : Theta-2Theta
          scan range           :    5.0000 - 85.0000 (deg)
          scan mode            : Continuous Scan
          scan speed           :    2.0000 (deg/min)
          sampling pitch       :    0.0200 (deg)
          preset time          :    0.60 (sec)

# Data Process Condition
    Smoothing                  [ AUTO ]
          smoothing points     : 51
    B.G.Subtruction            [ AUTO ]
          sampling points      : 51
          repeat times         : 30
    Ka1-a2 Separate            [ MANUAL ]
          Ka1 a2 ratio         : 50 (%)
    Peak Search                [ AUTO ]
          differential points  : 43
          FWHM threhold        : 0.050 (deg)
          intensity threhold   : 30 (par mil)
          FWHM ratio  (n-1)/n  : 2
    System error Correction    [ NO ]
    Precise peak Correction    [ NO ]
```

< Group: CA-IQ   Data: P210509_AM-1076-21 >

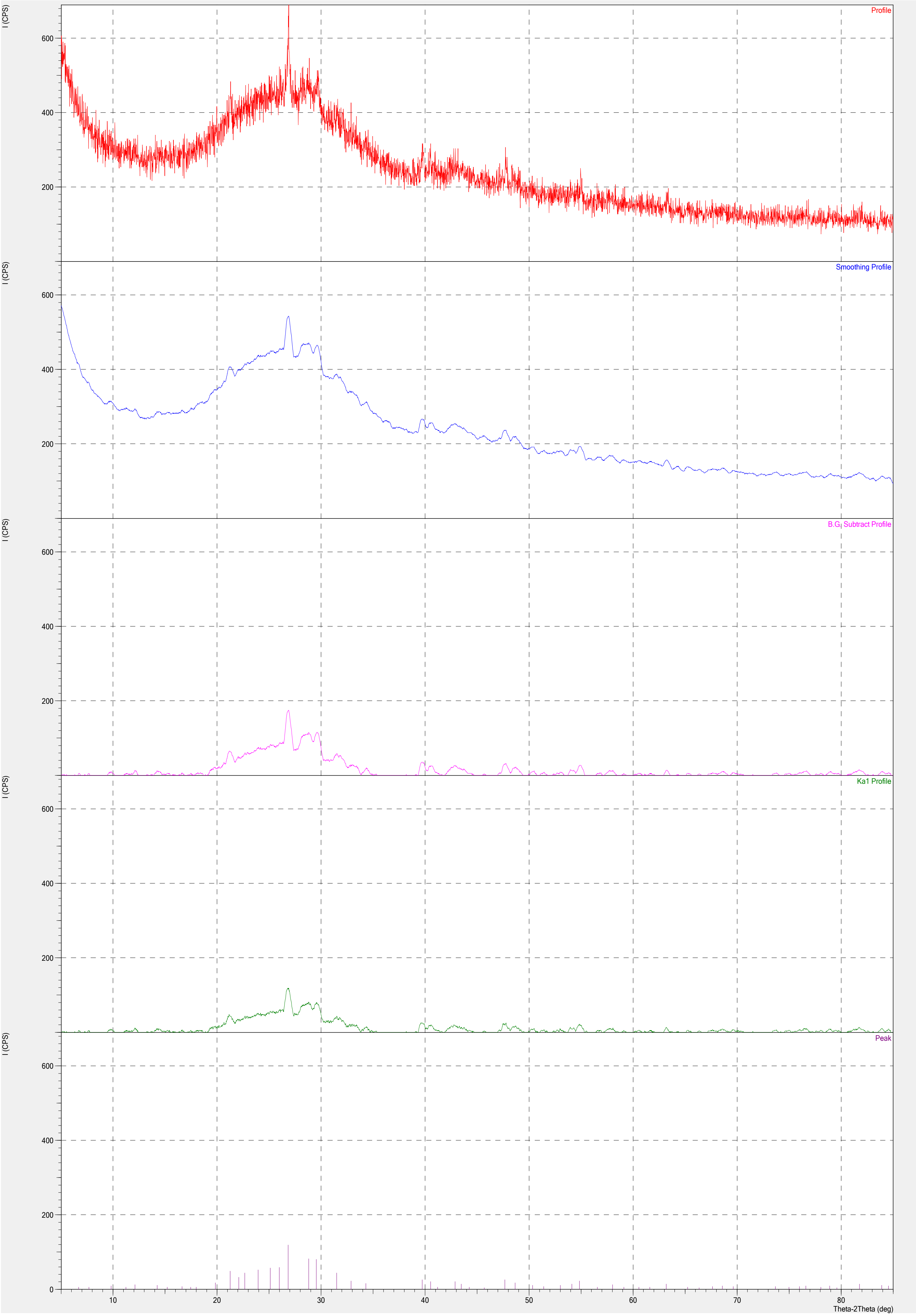

Supplement: Supplementary file 1 — Supplementary Information. [file 41598_2022_10165_MOESM1_ESM.pdf]
